# Evaluation of Antioxidant and Immunity-Enhancing Activities of *Sargassum pallidum* Aqueous Extract in Gastric Cancer Rats

**DOI:** 10.3390/molecules17078419

**Published:** 2012-07-11

**Authors:** Rui-Li Zhang, Wen-Da Luo, Tie-Nan Bi, Shen-Kang Zhou

**Affiliations:** 1 Department of Gastrointestinal Surgery, Taizhou Hospital of Zhejiang Province, Wenzhou Medical College, Linhai 317000, China; Email: rlzhanglhd@163.com (R.-L.Z.); tnbilhd@163.com (T.-N.B.); skzhoulhd@163.com (S.-K.Z.); 2 Division of Hematology and Medical Oncology, Taizhou Hospital of Zhejiang Province, Wenzhou Medical College, Linhai 317000, China

**Keywords:** *Sargassum pallidum* aqueous extract, IL-2, IL-4, TNF-α, antioxidant

## Abstract

The effect of *Sargassum pallidum* (brown seaweed) aqueous extract on the immunity function and antioxidant activities in was studied gastric cancer rats. Treatment with *Sargassum pallidum* aqueous extract at oral doses 400, 600 or 800 mg/kg body weight was found to provide a dose-dependent protection against *N*-methyl-*N′*-nitro-*N*-nitrosoguanidine (MNNG)-induced immunity damage and oxidative injury by enhancing serum interleukin-2 (IL-2), interleukin-4 (IL-4), interleukin-10 (IL-10) levels, decreasing interleukin-6 (IL-6), interleukin-1β (IL-1β), tumor necrosis factor-alpha (TNF-α) levels, preserving normal antioxidant enzymes activities, and by inhibiting lipid peroxidation in gastric mucosa. It can be concluded that *Sargassum pallidum* aqueous extract may enhance the immunity and antioxidant activities in gastric cancer rats.

## 1. Introduction

Gastric cancer is the second commonest cause of death from malignant disease worldwide [1]. Antioxidant compounds, such as vitamin C and vitamin E, play a key role in the prevention and termination of development of gastric cancer [2]. Gastric ulcer therapy faces a major drawback nowadays due to the unpredictable side effects of the long-term use of commercially available drugs. As it affects 5% of the global population [3], the treatment of this painful disease and its prevention has become one of the challenging medical problems of the day. It is shown that toxic oxygen radicals play an important role in the etiopathogenesis of gastric damage [4]. There are enzymatic and non-enzymatic defense mechanisms against toxic radicals which cause damage in tissues [5,6]. It was shown that in parallel to tissue damage there is a decrease in antioxidants such as glutathione (GSH) and superoxide dismutase (SOD) and an increase in oxidants such as malondialhehyde (MDA) and myeloperoxidase (MPO) [4]. Hence, the search is still on to find drug possessing antioxidant and antiulcer properties, which will serve as a powerful therapeutic agent to cure gastric ulceration, and the search extends to the systematic development of natural products.

Marine algae, including edible varieties, have been shown to produce potent antioxidant compounds [7,8,9,10]. Seaweeds have seen an emerging interest in the biomedical area, mainly due to their contents of bioactive substances which show great potential as anti-inflammatory, antimicrobial, antiviral, and anti-tumor drugs [11,12]. *Sargassum pallidum*, a brown seaweed widely distributed in the Chinese Yellow Sea and East China Sea, is rich in vitamins, amino acids, dihomogammalinolenic acid, trace elements and polysaccharides [13,14]. The anti-tumour and antioxidant activities of *Sargassum pallidum* have been reported [15,16]. In the present study, we investigated the effect of *Sargassum pallidum* aqueous extract on the immunity functions and antioxidant activities in gastric cancer rats.

## 2. Results

**Figure 1:**
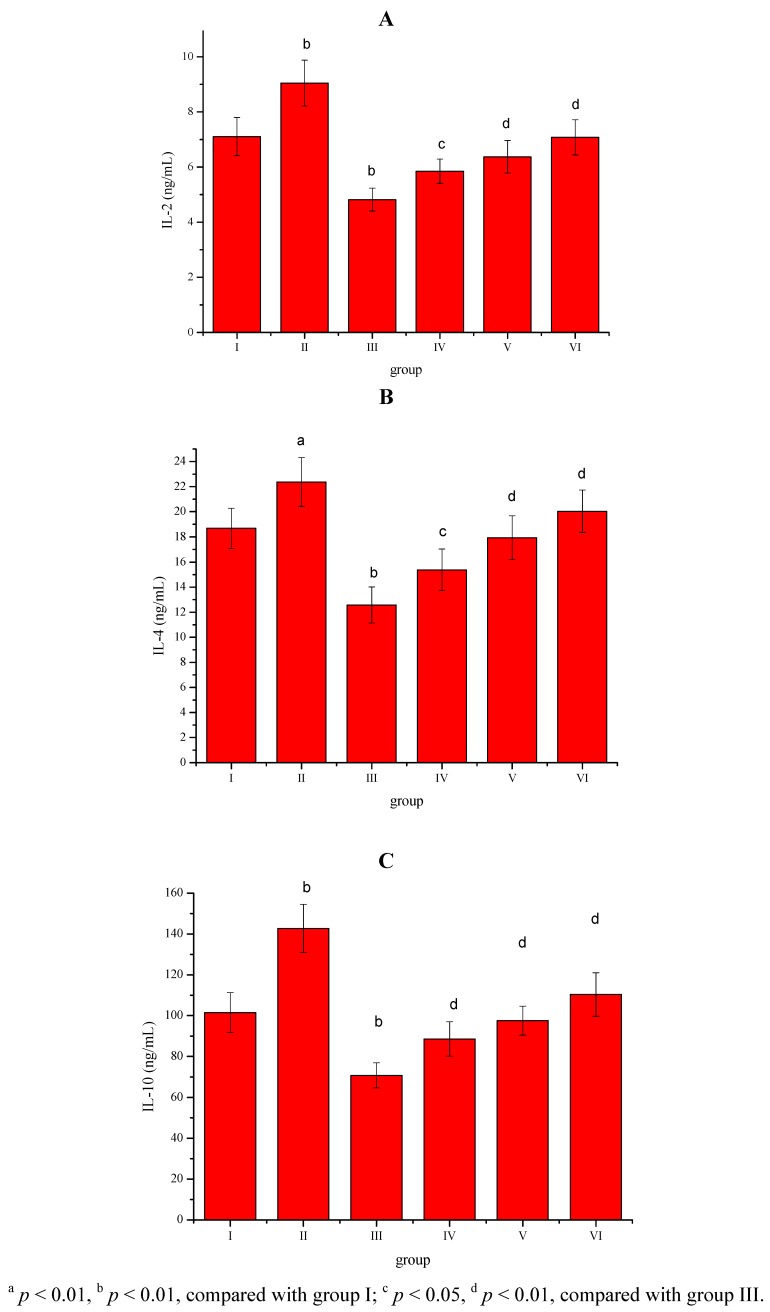
(**A**). Effect of *Sargassum pallidum* aqueous extract on serum IL-2, (**B**) IL-4 and (**C**) IL-10 levels in different groups.

The effect of the *Sargassum pallidum* aqueous extract (400, 600 and 800 mg/kg body weight) administrated to rats for 8 weeks on the serum IL-2, IL-4 and IL-10 levels is shown in Figure 1A–C. Compared with group I, serum IL-2, IL-4 and IL-10 levels in group II were significantly (*p* < 0.05; *p* < 0.01) increased, whereas serum IL-2, IL-4 and IL-10 levels in group III were significantly (*p* < 0.01) decreased. 

Compared with group III, *Sargassum pallidum* aqueous extract (400, 600 and 800 mg/kg body weight) treatment significantly (*p* < 0.05; *p* < 0.01) enhanced serum IL-2, IL-4 and IL-10 levels in groups IV, V and VI in a dose-dependent manner.

**Figure 2:**
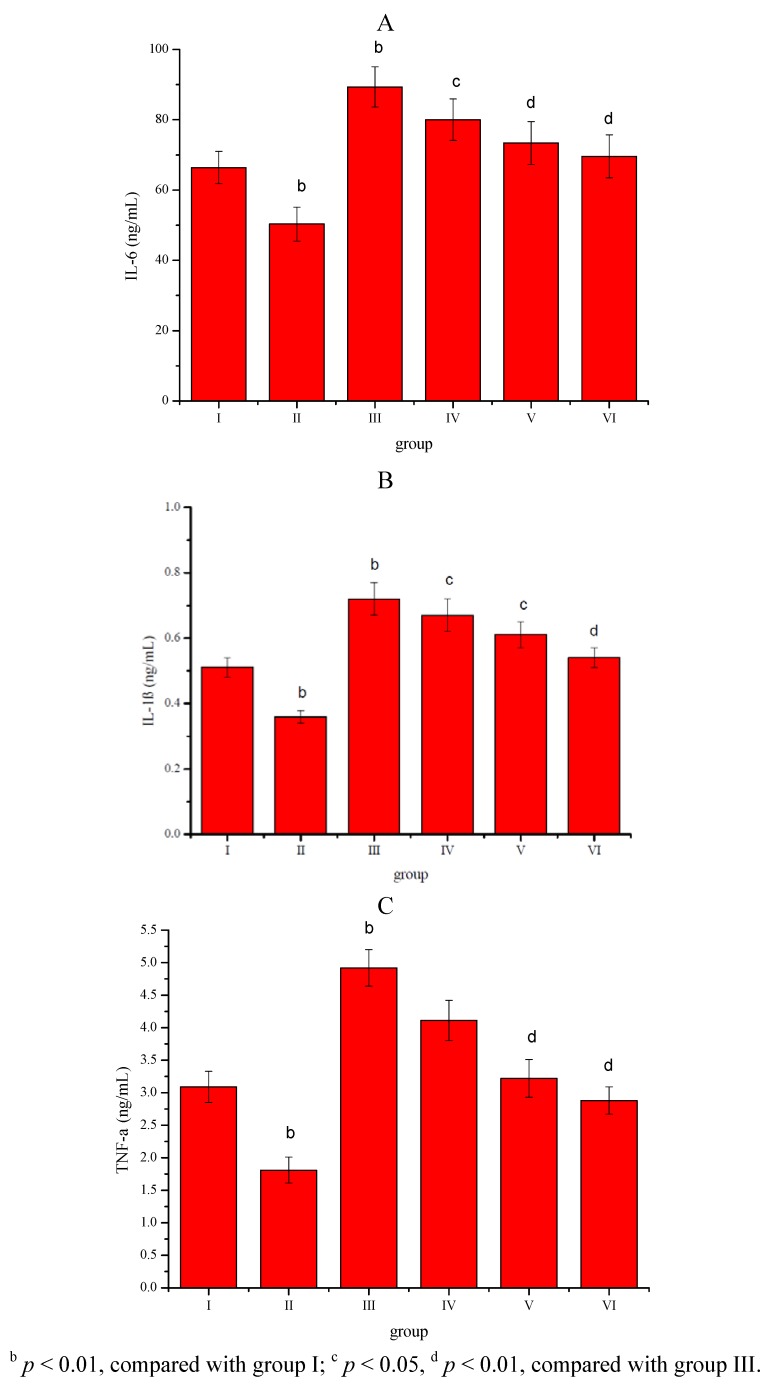
(**A**) Effect of *Sargassum pallidum* aqueous extract on serum IL-6, (**B**) IL-1β and (**C**) TNF-α levels in different groups.

The effect of the *Sargassum pallidum* aqueous extract (400, 600 and 800 mg/kg body weight) administrated to rats for 8 weeks on the serum IL-6, IL-1β and TNF-α levels is shown in Figure 2 A, B and C. Compared with group I, serum IL-6, IL-1β and TNF-α levels in group II were significantly (*p* < 0.01) decreased, whereas serum IL-6, IL-1β and TNF-α levels in group III were significantly (*p* < 0.01) increased. Compared with group III, *Sargassum pallidum* aqueous extract (400, 600 and 800 mg/kg body weight) treatment dose-dependently and significantly (*p* < 0.05; *p* < 0.01) decreased serum IL-6, IL-1β and TNF-α levels in group IV, V and VI.

**Figure 3:**
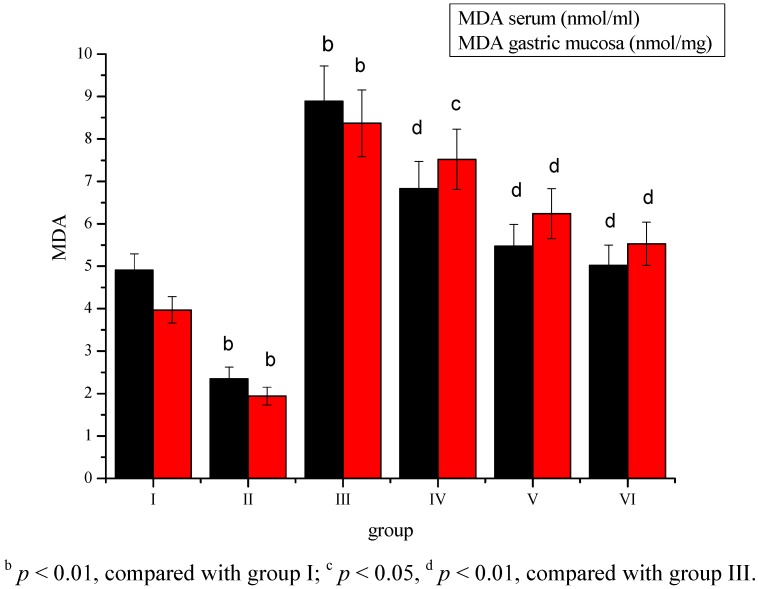
Effect of *Sargassum pallidum* aqueous extract on serum and gastric mucosa MDA level in different groups.

The level of MDA was significantly (*p* < 0.01) decreased in group II, while an increase in the MDA content was observed in the serum and gastric mucosa tissue of group III compared to group I (Figure 3). *Sargassum pallidum* aqueous extract administration significantly (*p* < 0.05; *p* < 0.01) decreased the concentration of serum and gastric mucosa MDA in a dose-dependent way in groups IV, V and VI.

**Figure 4:**
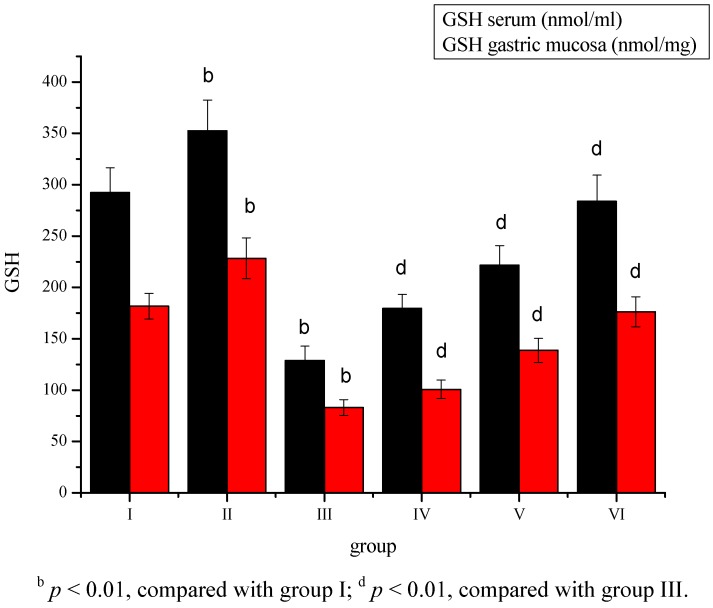
Effect of *Sargassum pallidum* aqueous extract on serum and gastric mucosa GSH level in different groups.

The level of GSH was significantly (*p* < 0.01) increased in group II, while a decrease in the GSH content was observed in the serum and gastric mucosa tissue of group III compared to group I (Figure 4). *Sargassum pallidum* aqueous extract administration significantly (*p* < 0.01) increased the concentration of serum and gastric mucosa GSH in a dose-dependent way in group IV, V and VI.

**Figure 5:**
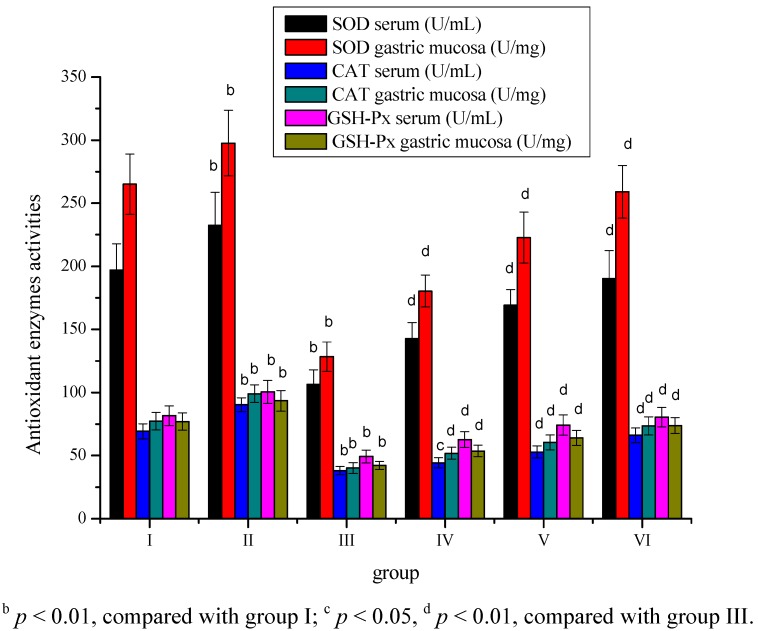
Effect of *Sargassum pallidum* aqueous extract on serum and gastric mucosa SOD, CAT and GSH-Px in different groups.

The activities of antioxidant enzyme were significantly (*p* < 0.01) increased in the group II, while a decrease in the antioxidant enzyme activities was observed in the serum and gastric mucosa tissue of group III compared to group I (Figure 5). *Sargassum pallidum* aqueous extract treatment dose-dependently and significantly (*p* < 0.01) elevated serum and gastric mucosa antioxidant enzyme activities in serum (Figure 5).

## 3. Discussion

The present study was undertaken to assess the anti-tumor effects of *Sargassum pallidum* aqueous extract on the immunity activity and oxidative injury in a gastric cancer rat model. We found that *Sargassum pallidum* aqueous extract led to significant suppression of serum IL-6, TNF-α levels, and significant enhancement of serum IL-2, IL-4, IL-10 levels in this model. 

One form of inflammatory cytokines is known as the pro-inflammatory polypeptide regulators. These cytokines (IL-6, IL-1β and TNF-α) are created primarily by immune cells that are engaged in the process of amplifying inflammatory reactions as a means of dealing with some sort of health threat to the body. By relaying messages between the cells, these cytokines help to trigger the immune system’s rate of response to whatever threat is present. Along with the pro-inflammatory cytokines, there is also anti-inflammatory cytokines (IL-2, IL-4, IL-10). These have the opposite effect, in that they help to limit of inflammation present. This means that both types of cytokines often work to balance each other as they stimulate cell production and effect communication between cells. Because of this close relationship, many researchers tend to downplay the distinction of each type of inflammatory cytokines, since both types can be simultaneously working on the same cell at any given point in time. There is increasing evidence suggesting that locally produced pro-inflammatory cytokines, such as IL-1, IL-6, IL-8 and TNF-α, are involved in normal physiological processes, as well as in regulating mucosal immune responses and thus should be involved in IBDs [17]. Besides many biological activities, both IL-1β and TNF-α are involved in the induction of inflammation, injury and carcinogenesis in a variety of tissues including the gastric mucosa [18,19,20]. Moreover, these cytokines were recently implicated in the mechanism ischemia–reperfusion injury progressing into gastric ulcer [21] and to mediate the delay in ulcer healing induced by H. pylori and its water extract [22,23]. Our present results suggested that *Sargassum pallidum* aqueous extract could decrease the inflammatory response and improve immunity function partly through stimulating inflammatory cytokines (IL-2, IL-4, IL-10) production and inhibiting pro-inflammatory cytokines production.

In an experiment conducted by Naito *et al*., the role of reactive oxygen species (ROS) in the etiopathogenesis of indomethacin-induced gastric damage was shown [24]. Against these detrimental effects of ROS, in tissues, enzymatic and non-enzymatic defense mechanisms were produced [25,26,27]. Tissue damage starts with lipid radical formation in the cell membrane. This radical first turns into lipid hydroperoxide, and then the damage is completed by the formation of toxic products, such as aldehyde, alkane, and malondialdehyde [24]. A decrease in ulcer areas due to chronic indomethacin administration means that this may trigger antioxidant activity in gastric tissue.

In the present study, serum and gastric mucosa MDA level in gastric cancer rats were significantly increased, whereas GSH level and antioxidant enzymes activities were significantly decreased. The results indicate that oxidative injury had happened to gastric cancer rats. The increase in the levels of TBARS indicates an enhanced lipid peroxidation leading to tissue injury and failure of the antioxidant defence mechanisms to prevent the formation of excess free radicals [28]. GSH and other antioxidants (*i.e.*, melatonin, vitamins) prevented tissue damage by keeping ROS levels in physiologic concentrations [29,30]. The protective action of antioxidant may be due to an inhibition of reactive oxygen species (ROS) inducing a chain reaction mediated by several antioxidant enzymes including SOD, GSH-Px and catalase. In the current study, the significant decrease in serum, gastric mucosa MDA level and increase in serum, gastric mucosa GSH level, SOD, catalase (CAT) and glutathione peroxidase (GSH-Px) activities after *Sargassum pallidum* aqueous extract treatment were observed in group IV–VI rats. This indicated that *Sargassum pallidum* aqueous extract could significantly decreased oxidative injury in gastric cancer rats. 

## 4. Experimental

### 4.1. Materials

*Sargassum pallidum* was purchased from Dongtou Hongda Marine Algae Ltd, Dongtou, China.

### 4.2. Preparation of *Sargassum pallidum* Aqueous Extract

*Sargassum pallidum* (200 g) was suspended in distilled water (2,000 mL), and then heated and boiled under reflux for 60 min. The decoction obtained was filtered, and the filtrate frozen at −70 °C and then lyophilised. The average yield of the lyophylised material (*Sargassum pallidum* aqueous extract) was approximately 15% (w:w). It was stored at ambient temperature until further use.

### 4.3. Treatment of Animals

Male Wistar rats (4 weeks old) weighing 85–100 g were purchased from the animal center of Wenzhou Medical College, China. The rats were housed five per cage in a room with controlled temperature and humidity. After 1 week of acclimatization, rats were randomly divided into six groups (10 rats per group): Group I, group II, group III, group IV, group V and group VI.

Group I served as the normal control and was given distilled water orally for the entire experimental period.

Group II received a basal diet and was treated with *Sargassum pallidum* aqueous extract (800 mg/kg body weight, dissolved in distilled water) alone for 8 weeks from week 26.

The remaining animals (groups III-VI) were given MNNG (25 mg/mL; Aldrich Chemical Co. Ltd, Milwaukee, WI, USA) in drinking water for 25 weeks and regular chow pellets over the entire study period. The MNNG was dissolved in deionized water at a concentration of 0.5 mg/mL and kept in a cool (4 °C), dark place. Just before use, the stock solution was diluted to 25 mg/mL with tap water. Rats were given MNNG solution from a bottle covered with aluminum foil to prevent photolysis of MNNG; the solution was replenished every other day. From Week 26, the rats had access to ordinary tap water from an automatic watering system. Then, group III served as the model control and was given distilled water orally for 8 weeks. Groups IV, V and VI were treated with *Sargassum pallidum* aqueous extract (400, 600 and 800 mg/kg body weight, dissolved in distilled water, respectively) for 8 weeks.

The experiment was terminated in the 33th week, and all rats were killed by cervical dislocation after an overnight fast. Blood was collected, and the plasma separated was used for analysis. Stomachs were excised to prepare a 10% homogenate for biochemical measurements.

### 4.4. Biochemical Measurements

Serum IL-2, IL-4, IL-10, IL-6, and TNF-α were measured using commercially available ELISA kits (Shanghai BlueGene Biotech CO., LTD). We followed the manufacturer's instructions to determine these biochemical indexes. Lipid peroxidation was estimated by measuring thiobarbituric acid-reactive substances (TBARS) and expressed in terms of malondialdehyde (MDA) content, according to the method of Draper and Hadley [31]. The MDA values were calculated using 1,1,3,3-tetraethoxypropane as standard and expressed as nmol of MDA/mL or mg.

Glutathione (GSH) was measured following the method of Fukuzawa and Tokumura [32]. Briefly supernatant (200 μL) was added to 0.25 M sodium phosphate buffer (1.1 mL, pH 7.4) followed by the addition of DTNB (130 μL, 0.04%). Finally, the mixture was brought to a final volume of 1.5 mL with distilled water and absorbance was read in a spectrophotometer at 412 nm.

SOD activity was determined with SOD Assay Kit A001 (Institute of Biological Engineering of Nanjing Jianchen, Nanjing, China). Superoxide was generated in xanthing oxidase and hypoxanthine, and the superoxide scavenging effect of serum and tissue was determined according to Oyanagui’s method [33]. Fifty percent inhibition was defined as one unit of SOD activity. 

The activity of catalase was determined by a commercial kit (Nanjing Jiancheng Company, Nanjing, China). Ammonium molybdate can terminate the decomposition reaction of H_2_O_2_ catalyzed by catalase. The surplus H_2_O_2_ may have an interaction with ammonium molybdate generating a kind of comoles compound (peroxomolybdic acid complex) with a distinctive colour. The absorbance was measured optically at 405 nm, where it had its maximum absorbance. One unit of enzyme is defined as the amount of enzyme required to breakdown 1 μmol H_2_O_2_ per second. 

Glutathione peroxidase (GSH-Px) activity was assayed by spectrophotometry [34]. Glutathione peroxidase may catalyze the reaction of GSH and hydroperoxides. The activity of the enzyme could be evaluated by the consumption of GSH. The reaction was started by addition of 400 μL diluted sample. GSH may react with 5,5-dithiobis-(2-nitrobenzoic acid) (DTNB) forming a yellow product. The absorbance was measured optically at 422 nm.

### 4.5. Statistical Analysis

All results are expressed as mean ± S.E.M. Statistical analyses were performed using one-way analysis of variance (ANOVA). Significant differences were determined by Tukey’s *post hoc* test. *F* values for which *p* < 0.05 were regarded as statistically significant.

## 5. Conclusions

*Sargassum* *pallidum* aqueous extract can improve the immunity function and decrease oxidative injury in gastric cancer rats.
